# A Randomized Phase 1 Pharmacokinetic Study Comparing the Potential Biosimilar LRG201902 With Liraglutide (Victoza^®^) in Healthy Male Subjects

**DOI:** 10.3389/fphar.2020.610880

**Published:** 2021-01-29

**Authors:** Gang Mai, Lianlian Fan, Mupeng Li, Peiwen Zhang, Chunyan Gan, Qian Huang, Jianzhong Shentu

**Affiliations:** ^1^ Phase 1 Clinical Trial Center, People’s Hospital of Deyang City, Deyang, China; ^2^ Research Center of Clinical Pharmacy, State Key Laboratory for Diagnosis and Treatment of Infectious Diseases, The First Affiliated Hospital, Zhejiang University School of Medicine, Hangzhou, China

**Keywords:** biosimilar, liraglutide, pharmacokinetics, bioequivalence, safety

## Abstract

**Objective:** Pharmacokinetic (PK) similarity between biosimilar candidate LRG201902 and European Union-sourced liraglutide reference product (Victoza^®^) was evaluated. Safety and immunogenicity were also assessed.

**Methods:** This single-dose, randomized, open-label, 2-period crossover study (CTR20192342) was conducted in thirty-eight healthy adult male subjects. Volunteers were randomized 1:1 at the beginning to receive a single 0.6 mg dose of Victoza^®^ or LRG201902 by subcutaneous injection during the first period. Following 8 days washout period, all subjects received the alternate formulation during the second period. Blood samples were collected up to 72 h after administration. The primary pharmacokinetic endpoints were AUC_0–t_, AUC_0–∞_, and C_max_. Pharmacokinetic similarity was achieved if 90% confidence intervals (CIs) of the geometric mean ratios (GMRs) of AUC0-t, AUC_0–∞_, and C_max_ were within the range of 80–125%. Other pharmacokinetic parameters including T_max_, t_½_, and λ_z_ were also measured. Safety profile and immunogenicity data were collected from each subject.

**Results:** C_max_, AUC_0–t_, and AUC_0–∞_ were similar between the two groups. GMRs of Cmax, AUC_0–t_, and AUC_0–∞_ were 113.50%, 107.21%, and 106.97% between LRG201902 and Victoza^®^ respectively. The 90% CIs for the GMRs of C_max_, AUC_0-t_, and AUC_0–∞_ were all within the PK equivalence criteria. Mean serum concentration-time profiles, secondary pharmacokinetic parameters (T_max_, t_½_, and λ_z_) were comparable between groups. Treatment-related adverse events were reported by 27.8% and 23.7% subjects in the LRG201902 and Victoza^®^ arms, respectively. All post-dose samples were detected negative for anti-drug antibodies.

**Conclusion:** This study demonstrates pharmacokinetic similarity of LRG201902 to Victoza^®^ in healthy subjects. The safety and immunogenicity profiles were similar for the two products.

## Introduction

Liraglutide is a glucagon like peptide-1 (GLP-1) receptor agonist analogue with 97% homology to human GLP-1. It stimulates pancreatic GLP-1 receptor to increase glucose-dependent insulin secretion, delay gastric emptying, and increase satiety (Knudsen and Lau., 2019). Liraglutide (Victoza®) was approved as an adjunct therapy to diet and exercise for management of type 2 diabetes (T2DM) in adults by the European Medicines Agency, 2009 and by the US Food and Drug Administration (FDA) in 2010. Based on results from the global ELLIPSE trial (Tamborlane et al., 2019), FDA approve Victoza® for the treatment of type 2 diabetes in children and adolescents aged 10–17 years in 2019. Several clinical trials repeatedly revealed the efficacy of liraglutide to induce weight loss (Davies et al., 2015; Pi-Sunyer et al., 2015). As a result, liraglutide (Saxenda®) was approved in the United States and Europe as an adjunct to a reduced-calorie diet and increased physical activity for weight management in adult patients in 2014 and 2015, respectively. Recently, liraglutide plus lifestyle therapy was found to significantly lower BMI standard-deviation score in adolescents with obesity (Kelly et al., 2020). Furthermore, liraglutide was demonstrated to reduce the risk of major adverse cardiovascular events in patients with T2DM (Marso et al., 2016) and Victoza® has been approved to prevent cardiovascular events in adults with T2DM. Also, liraglutide reduced the risk of the composite renal outcome in patients with T2DM (Mann et al., 2017). Currently, liraglutide has been recommend by clinical practice guidelines (American Diabete Association 2020a; American Diabete Association 2020b; Cosentin et al., 2020).

Despite significant therapeutic improvement for T2DM, biologic therapies are costly, limiting patient access to treatment. The availability of new biosimilar products, which have lower costs than reference biologicals, provides a potential means to overcome cost barriers to some degree and thus enhance access across the globe. A biosimilar is a biological product highly similar to an already licensed biologic product (the reference product) and expected to have similar quality, clinical efficacy and safety profiles, as stepwisely determined by comprehensive comparability assessments. The guidance for the development and approval of biosimilars has been clearly established by regulatory authorities, including the EMA (European Medicines Agency, 2014), US FDA (US Food and Drug Administration, 2015), and China National Medical Products Administration (NMPA) (China National Medical Products Administration, 2015). Recently, NMPA issued an additional directive guidance for liraglutide biosimilars (China National Medical Products Administration, 2020).

Several proposed liraglutide biosimilars are in different stages of development, some are in phase I (www.chinadrugtrials.org.cn identification number CTR20201785) or Phase III clinical trial (ClinicalTrials.gov identification number NCT03421119; www.chinadrugtrials.org.cn identification number CTR20201453, CTR20201449, CTR20201274, CTR20200400, CTR20200348, CTR20192168, CTR20190791, and CTR20190444). LRG201902 is being developed as a medicine that may prove to be biosimilar to Victoza®. The strength of LRG201902 is the same as that of the Victoza® dosage form that was originally approved. In preclinical studies, LRG201902 was shown to be highly similar to liraglutide in the European Union (EU) with respect to structure and *in vitro* biological activity (Unpublished data). The primary objective of the current study was to evaluate and compare the pharmacokinetic (PK) profiles of LRG201902 and EU-sourced reference liraglutide in healthy subjects. The secondary objectives were to assess additional PK parameters, safety and immunogenicity of LRG201902 and reference liraglutide in these subjects.

## Materials and Methods

### Investigational Products

LRG201902 (batch number 201906501) was sourced from Jiangsu Wanbang Biopharmaceuticals Co., Ltd. (Xuzhou, Jiangsu Province, China) and Victoza® (batch number HVGM816-2) was sourced from Novo Nordisk A/S (Bagsvaerd, Denmark). LRG201902 was supplied in a borosilicate glass barrels for pen-injectors and Victoza® was supplied in a pre-filled, multi-dose pens with both 3 ml of solution containing 18 mg liraglutide.

### Study Design and Ethics

This single-center study (www.chinadrugtrials.org.cn identification number CTR20192342) was approved by the local investigational review board and conducted in compliance with the provisions of the Declaration of Helsinki, the China’s current Good Clinical Practice (GCP), and the International Conference on Harmonization E6 Guidelines on GCP. Written informed consent was obtained from each participant at the screening visit prior to the initiation of any study-specific procedures.

Screening occurred within 7 days prior to dosing. Eligible subjects were admitted to the clinical research unit (CRU) on the day before dosing. Following an overnight fast at least 10 h, subjects were randomized to receive a single subcutaneous injection of 0.6 mg LRG201902 or Victoza® in a 1:1 ratio in the morning on day 1. Randomization codes were generated using SAS® version 9.4 (SAS Institute Inc., United States) before the study began.

Subjects remained in the study center for at least 72 h after dosing for PK and safety evaluations. They were discharged on Day 4 after the 72 h evaluations were completed. Subjects returned to the CRU on Day 8 and remained until Day 12 (end-of-study visit) for evaluation of safety, collection of PK samples. Subjects were monitored throughout the study for adverse events (AEs), vital signs, clinical laboratory results, and concomitant medication use.

### Study Population

Eligible subjects were healthy males aged between 18 and 45 years, with a body mass index of 19.0–26.0 kg/m^2^ and a total body weight >50 kg. Health was determined based on results of a medical history, physical examinations (including vital sign measurements), laboratory analysis (hematology, biochemistry, hepatic function tests, and urinalysis), and 12-lead ECGs conducted at screening.

Subjects were excluded if medical examinations revealed clinically significant abnormalities or any evidence or history of clinically significant disease. These included circulatory, respiratory, digestive, urinary, hematological, nervous, mental, endocrine, metabolic, and musculoskeletal systems. Subjects were ineligible for trial entry if they had a history of allergy, syncope or amaurosis, hypoglycemia, blood-injection-injury phobia; and family history of hereditary diseases. Subjects were also excluded if they had used any prescription or non-prescription medications or dietary supplements within 14 days, underwent intensive physical exercise or took in any food or drink containing caffeine or xanthine within 48 h prior to dosing. Tobacco smokers were not eligible for the study, nor were subjects exhibiting evidence of alcohol and/or substance abuse. Subjects who participated in trials of other investigational products within 3 months before or during administration of the study drug, individuals who donated blood, underwent massive blood loss, received blood products within 3 months before the study, subjects who received any surgical operation prior within 3 months before the study were also excluded. Subjects who have been vaccinated within the past 3 months, or plan to vaccination within 3 months after the last medication; those who unable to observe the dieting protocol of this trial; those with any clinically significant laboratory test results were also excluded. The subjects who planned to father a baby or sperm donation within 3 months after the last medication, those who don't willing to take effective non-pharmacological contraception were also excluded.

### Pharmacokinetic Evaluations

Serial blood samples (4 ml) for determination of plasma concentrations of liraglutide were collected by venous puncture or vein detained needle into K_2_EDTA tubes. Blood samples were collected within 1 h prior to initiation of liraglutide injection (predose) and at 1, 3, 5, 7, 8, 9, 10, 11, 12, 13, 14, 15, 16, 24, 36, 48, 60, and 72 h after injection. Blood samples were centrifuged for 10 min at 1700 g at 4°C. The supernatant plasma was transferred into two polypropylene storage tubes and stored at least −60°C until analysis.

Plasma concentrations of liraglutide were analyzed using a validated, sensitive, and specific liquid chromatography and tandem mass spectrometry (LC-MS/MS) assay conducted by Shanghai Xihua Scientific Co., Ltd. (Shanghai, China). Waters ACQUITY UPLC (Waters Corporation, Milford, Massachusetts) and AB Triple Quad 6500 + mass spectrometer (SCIEX Technologies, Framingham, Massachusetts) with electrospray ionization source were combined for the LC-MS/MS analysis. Liraglutide-Phenylalanine-^13^C9-^15^N provided by Wuxi Apptec (Shanghai) Co., Ltd. was used as an internal standard (IS). Chromatographic separation was achieved on a 2.1 × 50 mm, 1.7-µm Acquity BEH C18 column (Waters) at 40°C with a flow rate of 0.6 ml/min. The mobile phase A was 0.1% formic acid in water (v/v), and mobile phase B was 0.1% formic acid in acetonitrile (v/v). The method was validated for linear range, quantitative limit, accuracy, precision, recovery, selectivity, and stability. The quality control samples included in each assay were prepared in the same way to achieve final concentrations of 0.50, 1.50, 8.75, 75.0, and 120 ng/ml. Both the CVs for within-run and between-run precisions were less than 15%. And the within-run and between-run accuracy’s across the assay range were all within 100 ± 10%. The recovery were 74.3% and 97.2% for analyte and IS, respectively. The endogenous substances in blank plasma did not interfere with the determination of the analyte and IS. There were no interferences between analyte and IS. The mean IS normalized matrix factors (MFs) were 94.5%, 92.2%, and 99.8% at the high, medium, and low QC concentrations, respectively. The %CVs of IS normalised MFs at each QC level were less than 6%.

During analysis, PK samples before and after T_max_ in which plasma liraglutide concentrations were below the quantification limit (BQL) were listed as zero and missing value, respectively. All missing data was indicated with “-” or NA (not applicable) in the concentration data list. Any missing samples are indicated in “M”, and data without concentration due to insufficient plasma volume for reanalysis or other reasons specified in the laboratory process were indicated in “NR”.

The PK parameters assessed included maximum observed plasma concentration (C_max_), time at which C_max_ was observed (t_max_), AUC from zero to the time of the last quantifiable concentration (AUC_0–t_), AUC from zero extrapolated to infinity (AUC_0–∞_), terminal half-life (t_½_), and first-order rate constant of drug associated with the terminal portion of the curve (λ_z_).

### Immunogenicity Evaluations

Blood samples to detect anti-drug antibody (ADA) were collected day 1 pre-dose, 8 and 12 days after the first dose. A validated immunoassay was used to detect anti-bodies capable of binding LRG201902 or liraglutide (EU) by Shanghai Xihua Scientific Co., Ltd. (Shanghai, China). Any sample positive for binding ADAs was to be assessed for neutralizing anti-bodies capable of binding to LRG201902 or liraglutide (EU).

### Safety Evaluations

Subjects were monitored for AEs throughout the study. All observed or patient-reported adverse events (AEs) were assessed for severity and relationship to the study drug treatment using NCI Common Terminology Criteria for Adverse Events (CTCAE) Version 5.0. Other safety assessments included laboratory tests (hematology, chemistry, and urinalysis), physical examinations, vital signs, electrocardiograms, and finger-stick blood glucose.

### Statistical Methods

Sample size calculations were carried out using software PASS 16 (NCSS, Kaysville, Utah, United States), assuming that the pharmacokinetic parameters C_max_, AUC_0–t_, and AUC_0–∞_ were the primary endpoints. The coefficient of variations of pharmacokinetic parameters were predicted to be 22% on the basis of data obtained from a pilot study in healthy volunteers (Unpublished data) and report published by the EMA (European Medicines Agency, 2009). Thirty-two subjects were required to provide a power of at least 92.5% to demonstrate bioequivalence for each end point. This calculation was based on two one-sided *t*-test procedure with a type 1 error rate of 5% and an assumed a true ratio of 0.95. This procedure corresponded to the acceptance criteria for 90% confidence interval (CI). Assuming a 20% dropout rate, a sample size of 38 subjects was finally required.

The PK analysis used actual sample collection times. All parameters were calculated using standard non-compartmental methods (WinNonlin® Professional Network Edition, Version 8.1, Pharsight Corporation, St Louis, MO, United States) for all subjects with an evaluable LRG201902 or liraglutide plasma concentration versus time profile. Average bioequivalence method was used to evaluate the bioequivalence of two formulations of liraglutide. The point estimate and 90% CIs for ratio of the least square geometric means (GMs) for C_max_, AUC_0–t_, and AUC_0–∞_ were estimated using an analysis of variance with the sequence, period, treatment as fixed effects and subject within sequence as random effect. Pharmacokinetic equivalence was established if the 90% CIs for the ratio of least square GMs of primary PK parameters (C_max_, AUC_inf_, and AUC_last_) comparing LRG201902 versus liraglutide (EU) entirely fell within the standard equivalence criteria of 0.80 and 1.25. Other statistical analyses were conducted using SAS® version 9.4 (SAS Institute Inc., Cary, NC, United States). Prior to statistical modeling, PK parameters were log-transformed.

All subjects who received a complete dose of either LRG201902 or reference liraglutide, and from whom at least one post-treatment PK sample with a concentration above the lower limit of quantitation for liraglutide was collected, were to be included in the PK analysis population. The safety population comprised all randomized subjects who received any amount of investigational product. Safety analysis included descriptive summaries of AEs and the incidence of ADAs.

## Results

### Subjects

**FIGURE 1:**
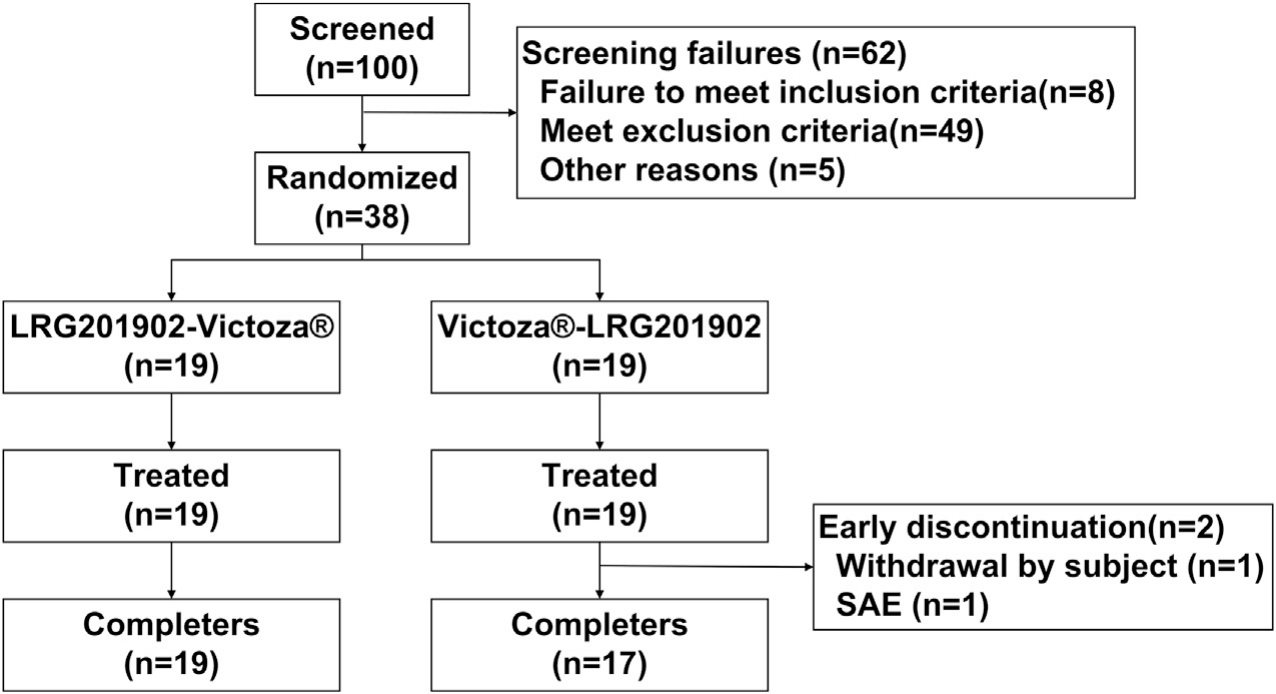
Study design and subject flow.

A total of 38 healthy male subjects were enrolled in the study and randomized in a 1:1 ratio to one of the two treatment sequences. Thirty-six subjects completed the study and two assigned to the Victoza®-LRG201902 sequence withdrew after completing the first period (one due to a serious adverse event of non-Hodgkin's lymphoma and one due to personal reason, Figure 1). Hence, all subjects were included in the safety set, pharmacokinetic concentration set, pharmacokinetic parameter set and bioequivalence set. The demographic and baseline characteristics of the subjects were comparable between the two treatment groups (Table 1).

**TABLE 1:** Demographic characteristics of all the subjects in the study.

Parameters	LRG201902-Victoza® (n = 19)	Victoza®-LRG201902 (n = 19)	Total (n = 38)
Age (years)			
Mean (SD)	25.5 (9.01)	23.1 (4.5)	24.3 (7.1)
Median (range)	21.0 (18–44)	22.0 (18–35)	22.0 (18–44)
Gender, n (%)			
Male	19 (100.0)	19 (100.0)	38 (100.0)
Female	0	0	0
Ethnicity, n (%)			
Han	19 (100.0)	18 (94.7)	37 (97.4)
Other	0	1 (5.3)	1 (2.6)
Height (cm)			
Mean (SD)	167.1 (6.0)	169.7 (3.9)	168.4 (5.2)
Median (range)	166.7 (156.2–176.0)	169.5 (162.5–181.1)	169.1 (156.2–181.1)
Weight (kg)			
Mean (SD)	61.6 (6.6)	63.9 (7.3)	62.7 (7.0)
Median (range)	60.2 (50.2–76.7)	63.9 (52.9–75.9)	62.6 (50.2–76.7)
BMI (kg/m^2^)			
Mean (SD)	22.1 (2.0)	22.2 (2.0)	22.1 (2.0)
Median (range)	21.4 (19.0–25.7)	21.9 (19.1–25.2)	21.6 (19.0–25.7)

### Pharmacokinetics

**FIGURE 2:**
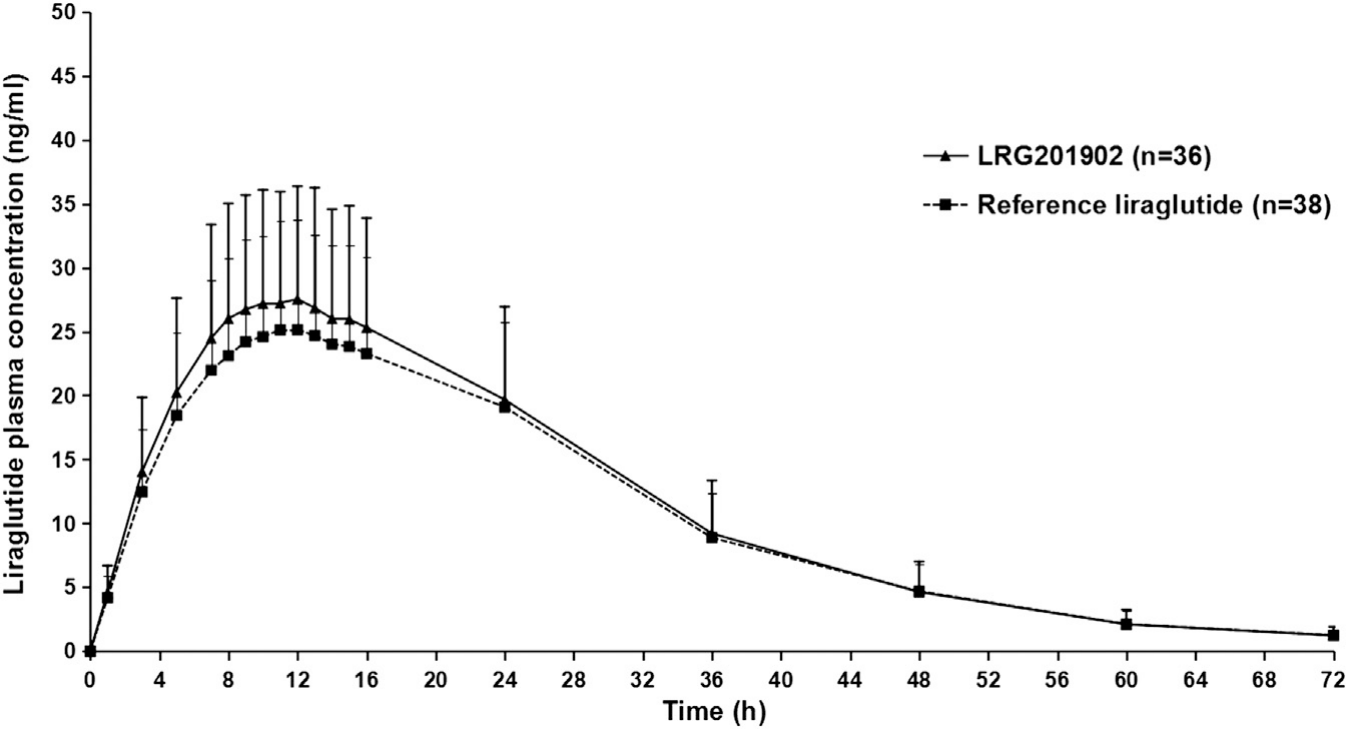
Mean (±SD) plasma concentration-time profiles of liraglutide after a single 0.6 mg subcutaneous administration of Victoza^®^ or LRG201902 in linear scale.

**FIGURE 3:**
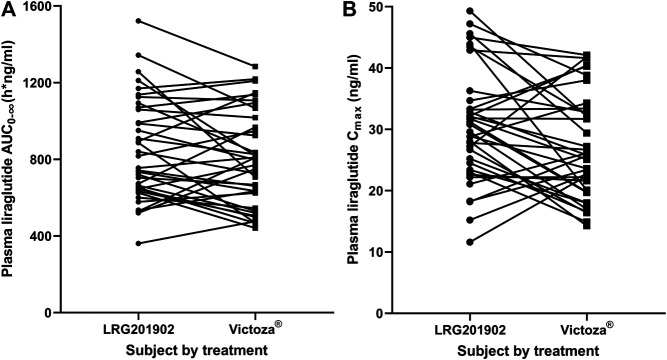
Stick plots comparing AUC_0-∞_ **(A)** and C_max_ **(B)** of individual subject following LRG201902 or Victoza^®^ treatment.

The LRG201902 and reference liraglutide exhibited a similar median plasma concentration-time profile following a single-dose subcutaneous injection (Figure 2). Figure 3 showed comparisons for AUC_0–∞_ and C_max_ of 36 subjects following LRG201902 or Victoza® treatment.

Consistent with the mean concentration-time profiles, the main and secondary pharmacokinetic parameters were comparable between treatment groups (Table 2). The geometric mean values of AUC_0–t_, AUC_0–∞_, and C_max_ for the LRG201902 were slightly higher than those for the reference liraglutide. Equivalence of LRG201902 and reference liraglutide in healthy male subjects was demonstrated, with ratios of least squares geometric means (90% CI) for AUC_0–t_, AUC_0–∞_, and C_max_ within the predefined range of 80–125% (Table 3).

**TABLE 2:** Pharmacokinetic parameters of LRG201902 and Victoza®.

PK parameter		LRG201902 (n = 36)	Victoza® (n = 38)
AUC_0-t_ (h*ng/ml)	Mean (SD)	830.47 (261.76)	783.44 (241.43)
	CV%	31.52	30.82
AUC_0-∞_ (h*ng/ml)	Mean (SD)	849.52 (267.58)	803.36 (248.71)
	CV%	31.5	30.96
C_max_ (ng/ml)	Mean (SD)	30.55 (9.23)	27.25 (8.51)
	CV%	30.21	31.22
T_max_ (h)	Median	12	11
	Range	7.0–24.0	7.0–24.0
t_1/2_ (h)	Mean (SD)	10.71 (1.95)	11.01 (1.99)
	CV%	18.19	18.03
λ_z_ (1/h)	Mean (SD)	0.067 (0.013)	0.065 (0.011)
	CV%	19.66	17.10

SD, standard deviation; CV, coeffificient of variation.

**TABLE 3:** Bioequivalence Statistics of pharmacokinetic parameters.

PK parameter (unit)	Geometric means		GM ratio (%)^a^	90% CI (%)	CV (%)	Power (%)
	LRG201902 (n = 36)	Victoza® (n = 38)				
AUC_0-t_ (h*ng/ml))	800.88	746.99	107.21	100.28, 114.63	16.95	98.44
AUC_0-∞_ (h*ng/ml)	819.08	765.69	106.97	100.00, 114.43	17.07	98.55
C_max_ (ng/ml)	29.47	25.97	113.50	104.70, 123.04	20.56	63.17

GM, geometric means; CV, coeffificient of variation.

^a^ Test-to-reference ratio of adjusted geometric means.

### Safety

Overall safety profiles were similar for both LRG201902 and reference liraglutide, and both agents were well tolerated. In total, 35 treatment-emergent adverse events (TEAEs) were reported: 10 subjects (27.8%) reported 21 TEAEs in the LRG201902 group, compared with nine subjects (23.7%) reporting 14 TEAEs in the reference liraglutide group. Hyperidrosis, dizziness and malaise were the most common TEAEs, reported by three subjects (8.3%) in the LRG201902 group. Also, dizziness was the most common TEAE reported by three subjects (7.9%) in the reference liraglutide group (Table 4). A total of 12 subjects reported a TEAE considered related to study drug: eight subjects in the LRG201902 group, compared with four subjects in the reference liraglutide group (Table 4). Rates of hyperidrosis (8.3% vs 2.6%), dizziness (8.3% vs 2.6%), malaise (8.3% vs 0), serum potassium increased (5.6% vs 0), serum thyroid stimulating hormone increased (2.8% vs 0), toothache (2.8% vs 0), and urinary frequency (2.8% vs 0) were numerically higher in the LRG201902 group compared with the reference liraglutide group.

**TABLE 4:** Summary of treatment-emergent adverse events.

MedDRA preferred term	LRG201902 (n = 36)	Victoza^®^(n = 38)	Total (n = 38)
Subjects with at least one TEAEs, n (%)	10 (27.8%)	9 (23.7%)	17 (44.7%)
Hyperidrosis^a^	3 (8.3%)	1 (2.6%)	4 (10.5%)
Erythrosis	2 (5.6%)	0	2 (5.3%)
Pruritus	1 (2.8%)	0	1 (2.6%)
Dizziness^a^	3 (8.3%)	3 (7.9%)^b^	5 (13.2%)^b^
Malaise^a^	3 (8.3%)	1 (2.6%)^b^	4 (10.5%)^b^
Paleness	0	1 (2.6%)	1 (2.6%)
Serum potassium increased^a^	2 (5.6%)	0	2 (5.3%)
Urine protein positive	1 (2.8%)	0	1 (2.6%)
Serum thyroid stimulating hormone increased^a^	1 (2.8%)	0	1 (2.6%)
Serum uric acid increased^a^	0	1 (2.6%)	1 (2.6%)
Hyperglycemia	1 (2.8%)	0	1 (2.6%)
Abdominal pain^a^	0	1 (2.6%)	1 (2.6%)
Diarrhea^a^	0	1 (2.6%)	1 (2.6%)
Abdominal distention^a^	0	1 (2.6%)	1 (2.6%)
Toothache^a^	1 (2.8%)	0	1 (2.6%)
Tinnitus^a^	0	1 (2.6%)	1 (2.6%)
Musculoskeletal discomfort	1 (2.8%)	0	1 (2.6%)
Epistaxis	0	1 (2.6%)	1 (2.6%)
Non Hodgkin’s lymphoma	0	1 (2.6%)	1 (2.6%)
Frequent urination^a^	1 (2.8%)	0	1 (2.6%)
Palpitation	0	1 (2.6%)	1 (2.6%)

TEAE, treatment-emergent adverse event.

^a^ TEAEs related to study drug.

^b^ The numbers and frequencies for dizziness and malaise related to Victoza® were 1 (2.6%) and 0, respectively. Accordingly, total numbers and frequencies for dizziness and malaise changed to 4 (10.5%) and 3 (7.9%).

In the reference liraglutide group, one subject experienced grade 2 paleness and dizziness and another subject experienced grade 3 non-Hodgkin's lymphoma (NHL) leading to discontinuation from the study. And the remaining 17 subjects reported TEAEs with grade 1 in severity. All TEAEs resolved by the end of the study, with the exception of a subject in the reference liraglutide group (NHL). There were no deaths or TEAEs of Grade 4 or higher. The SAE of NHL was considered not related to study drug.

### Immunogenicity

There were no preexisting binding ADAs detected in baseline samples and no subjects had a positive ADA test at the end of the study.

## Discussion

These data firstly demonstrates that the pharmacokinetics of LRG201902 and reference liraglutide were equivalent in healthy subjects, as measured by the primary PK endpoints AUC_0–t_, AUC_0–∞_, and C_max_. Secondary PK endpoints (T_max_, t_1/2_, and λ_z_) were also comparable between LRG201902 and reference liraglutide.

Both agents were well tolerated, with the safety profile of LRG201902 comparable to that of reference liraglutide. The most frequently occurred TEAEs were hyperidrosis, dizziness and malaise in LRG201902 group. Dizziness occurred in 7.9% of Victoza®-treated subjects. One subjects in Victoza® group developed a SAE of NHL, which was not related with Victoza® and not reported in previous studies (US Food and Drug Administration, 2017). A previous trial revealed that the most common adverse events were of gastrointestinal origin after multiple Victoza® administration in healthy Chinese male subjects (Jiang et al., 2011). Previous studies have reported that the most common adverse reactions, reported in ≥5% of patients treated with Victoza® were: nausea, diarrhea, vomiting, decreased appetite, dyspepsia, constipation (US Food and Drug Administration, 2017). Immunogenicity did not differ between treatment groups (none of the trial subjects exhibited a positive ADA test result) in the present study. Low titers of ADAs were detected in 8.6% of Victoza®-treated patients during the LEAD trials (Blonde and Russell-Jones, 2009). And in the LEADER trial, ADAs were detected in 11 out of the 1247 (0.9%) Victoza®-treated patients with antibody measurements (US Food and Drug Administration, 2017).

Biosimilars are expected to have minor structural differences from their reference product. The EMA, FDA and NMPA require that biosimilarity is demonstrated via a stepwise developmental approach that includes analytical, non-clinical and clinical data, with clinical evidence encompassing PK, efficacy, safety, and immunogenicity. The importance of conducting a direct, comparative PK study between a biosimilar and the relevant reference product is highlighted by these regulatory agencies. Thus, these data represent an important component of the regulatory information required for approval of LRG201902 in these countries and region. According to the NMPA guidelines, all biosimilars of liraglutide used in clinical trials have to be compared to the reference listed drug. Victoza® (EU-sourced) was approved by NMPA in 2011; therefore, it was chosen as the reference for this study. In common with most PK studies, this trial was conducted in healthy, male volunteers. Healthy subjects were used in this study to avoid the potentially high variability of liraglutide exposure that may occur in patients with T2DM.

There are no liraglutide biosimilar products in the market. Several liraglutide bisosimilar candidates are currently in clinical trials. In addition, three synthetic peptide products of generic liraglutide injection solution have been submitted as an abbreviated new drug application to NMPA (www.cde.org.cn identification number CYHS1900863, CYHS1900746, and CYHS1700556). Furthermore, two phase III trials of LRG201902 are ongoing in China, in which the efficacy and safety of LRG20190 for T2DM (CTR20201453) and adult overweight or obese (CTR20201449) are being compared with those of reference liraglutide. The results of the phase III studies will be reported in a separate communication.

The present study complies with regulatory requirements of biosimilar development and evaluated pharmacokinetic, safety and immunogenicity profiles in subjects with sufficient sample size. This study had several limitations, which should be considered. Only male subjects were included in this study. However, both male and female patients will use liraglutide in clinical practice. In addition, pharmacodynamic profile of liraglutide has not been well studied with no determination of plasma glucose and insulin. Only finger-stick blood glucose was measured before and up to 25 h after liraglutide administration. Furthermore, ADA detection was performed before and 8 and 12 days after the first liraglutide dose, which may influence the observed incidence of ADA.

In conclusion, this phase I study demonstrate that there were no differences between LRG20190 and reference liraglutide Victoza® with respect to PK profile, safety, and tolerability after a single subcutaneous injection. No new safety signals with regard to treatment with LRG20190 were identified and no subject tested positive for ADAs. In addition to the results of structural and functional characterization, these results provide further support that the proposed biosimilar LRG20190 is highly similar to EU-authorized liraglutide reference products.

## Data Availability Statement

The raw data supporting the conclusions of this article will be made available by the authors, without undue reservation.

## Ethics Statement

The studies involving human participants were reviewed and approved by Clinical Trial Ethics Committee, People’s Hospital of Deyang City. The participants provided their written informed consent to participate in this study.

## Author Contributions

All authors listed have made a substantial, direct, and intellectual contribution to the work and approved it for publication.

## Funding

The authors declare that this study received funding from Jiangsu Wanbang Biopharmaceuticals Co., Ltd. The funder was not involved in the study design, collection, analysis, interpretation of data, the writing of this article or the decision to submit it for publication. This study was also funded by Deyang Science and Technology Program (No.FY202003).

## Conflict of Interest

The authors declare that the research was conducted in the absence of any commercial or financial relationships that could be construed as a potential conflict of interest.
